# Replication of *Norovirus* in Cell Culture Reveals a Tropism for Dendritic Cells and Macrophages

**DOI:** 10.1371/journal.pbio.0020432

**Published:** 2004-11-30

**Authors:** Christiane E Wobus, Stephanie M Karst, Larissa B Thackray, Kyeong-Ok Chang, Stanislav V Sosnovtsev, Gaël Belliot, Anne Krug, Jason M Mackenzie, Kim Y Green, Herbert W. Virgin

**Affiliations:** **1**Department of Pathology and Immunology, Washington University School of MedicineSt. Louis, MissouriUnited States of America; **2**Laboratory of Infectious Diseases, National Institute of Allergy and Infectious Diseases, National Institutes of Health, Department of Health and Human ServicesBethesda, MarylandUnited States of America; **3**Sir Albert Sakzewski Virus Research Centre, Royal Children's Hospital, and Clinical Medical Virology Centre, University of QueenslandBrisbaneAustralia

## Abstract

Noroviruses are understudied because these important enteric pathogens have not been cultured to date. We found that the norovirus murine norovirus 1 (MNV-1) infects macrophage-like cells in vivo and replicates in cultured primary dendritic cells and macrophages. MNV-1 growth was inhibited by the interferon-αβ receptor and STAT-1, and was associated with extensive rearrangements of intracellular membranes. An amino acid substitution in the capsid protein of serially passaged MNV-1 was associated with virulence attenuation in vivo. This is the first report of replication of a norovirus in cell culture. The capacity of MNV-1 to replicate in a STAT-1-regulated fashion and the unexpected tropism of a norovirus for cells of the hematopoietic lineage provide important insights into norovirus biology.

## Abstract 2

Noroviruses -- which cause epidemic gastroenteritis -- can now be grown in cells of the innate immune system, providing a tool to examine this pathogen while offering insights into norovirus biology

## Introduction

Viruses within the genus *Norovirus* (formerly “Norwalk-like viruses”) of the family *Caliciviridae* are major agents of acute gastroenteritis (Green et al. 2001). Norovirus research, including the development of prevention and control strategies, has been hampered by the failure to grow these viruses in cultured cells despite extensive efforts (Duizer et al. 2004). Most noroviruses identified thus far have been associated with gastrointestinal disease in humans, but members of the genus have been found in other species as well (Green et al. 2001; Karst et al. 2003). Our recent discovery of the first murine norovirus, murine norovirus 1 (MNV-1), and demonstration of its ability to infect the intestinal tract of mice following oral inoculation provided an opportunity to analyze the pathogenesis of this norovirus in mice (Karst et al. 2003). This previous study demonstrated that the cellular transcription factor STAT-1 and interferon (IFN) receptors are critical for resistance to MNV-1 infection in vivo. The availability of MNV-1 and STAT1-deficient (STAT1^−/−^) mice (Durbin et al. 1996; Meraz et al. 1996) that are highly susceptible to MNV-1 infection allowed us to revisit efforts to develop a cell culture system for noroviruses.

Here we show for the first time that MNV-1 grows in macrophages (MΦ) and dendritic cells (DCs) and provide the first tissue culture model for a norovirus. Using this model we demonstrate that MNV-1 growth in vitro was inhibited by the IFN-αβ receptor and STAT-1. In addition, we isolated the first three-times plaque-purified strain of MNV-1 (MNV-1.CW1) and characterized it in vitro and in vivo. Sequencing of serial passages of MNV-1.CW1 indicated remarkable sequence stability over time and indicated that an amino acid substitution in the capsid protein of serially passaged MNV-1 was associated with a loss of virulence in vivo.

## Results/Discussion

### MNV-1 Replicates in Murine MΦ and DCs

**Figure 1:**
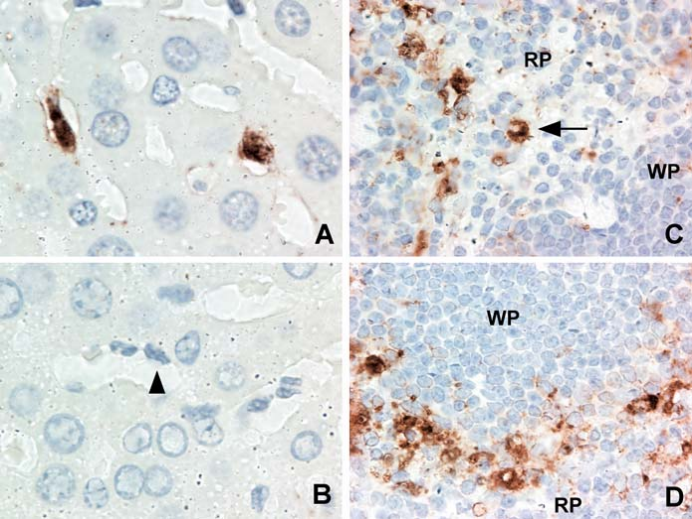
MNV-1-Specific Staining In Vivo Occurs in Cells of the MΦ Lineage Immunohistochemistry was performed on liver (A and B) and spleen (C and D) sections from STAT1^−/−^ mice 2 d after oral infection. MNV-1-specific staining was seen in Kupffer cells of infected livers when probed with MNV-1 immune (A) but not preimmune (B) serum. A selected Kupffer cell lining the sinusoid is indicated by an arrowhead. MNV-1-specific staining consistent with MΦ was seen in red pulp (C) and marginal zone (D) in the spleen. The arrow indicates a cell with MΦ morphology. No staining was observed in tissues from mice infected for 1 d, in infected tissues incubated with preimmune serum, or in mock-infected tissues incubated with immune serum. RP, red pulp; WP, white pulp.

As part of our ongoing investigation into MNV-1 pathogenesis, STAT1^−/−^ mice were infected with MNV-1 by the oral route and tissue sections analyzed by immunohistochemistry for the presence of MNV-1 protein. MNV-1-specific staining was observed in spleen and liver 2 d postinfection (Figure 1). Interestingly, in the liver, Kupffer cells (resident macrophages of the liver) lining the sinusoids were specifically stained by MNV-1 immune serum (compare Figure 1A and 1B). In the spleen, staining was found primarily in the red pulp and the marginal zone, but also in non-lymphoid cells within the white pulp (Figure 1C and 1D). This pattern is consistent with staining of MΦ and DCs (Metlay et al. 1990; Leenen et al. 1998). Furthermore, in some cases virus-antigen-positive MΦ were detected (Figure 1C).

**Figure 2:**
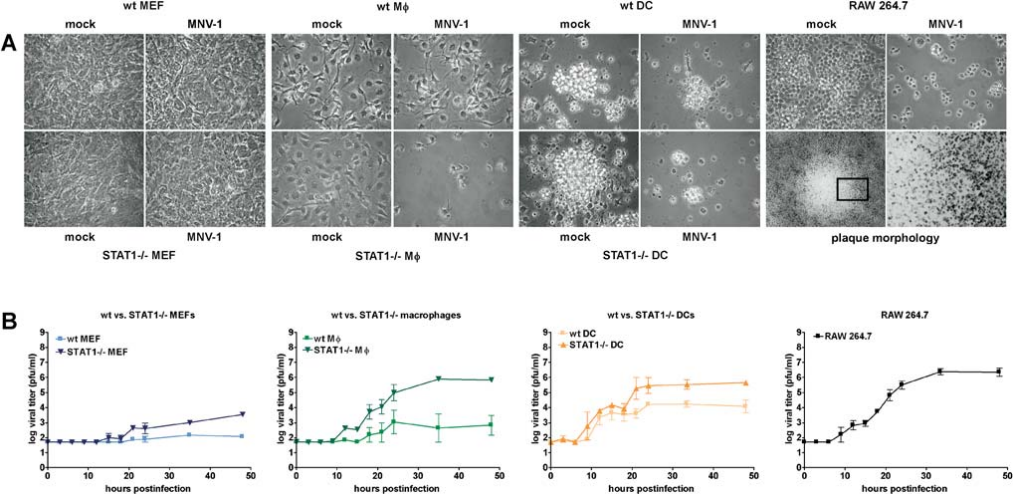
MNV-1 from Brain Homogenate Replicates in Cells of the DC and MΦ Lineage In Vitro BMDCs and BMMΦ, as well as MEFs from wt or STAT1^−/−^ mice, and RAW 264.7 cells were infected with a MOI of 0.05. (A) MNV-1 causes CPE in permissive cells. MNV-1- or mock-infected cells were observed by light microscopy 2 d postinfection. The boxed area is magnified further to show the border of the plaque. (B) Infected cell lysates were analyzed in two to four independent experiments by plaque assay at various timepoints postinfection to calculate standard deviations. For wt BMMΦ, MNV-1 growth was detected in two out of four experiments.

Because cells containing viral antigen in infected mice resembled MΦ, we examined whether cells of the hematopoietic lineage such as MΦ and DCs were permissive for MNV-1 replication in vitro. Bone-marrow-derived MΦ (BMMΦ) and bone-marrow-derived DCs (BMDCs) were inoculated with a MNV-1 stock derived from the brain of infected IFNαβγ receptor^−/−^ (IFNαβγR^−/−^) mice (Karst et al. 2003). Cytopathic effect (CPE) in cell monolayers was visible within 2 d in STAT1^−/−^ BMMΦ and BMDCs, but not STAT1^−/−^ murine embryonic fibroblasts (MEFs) (Figure 2A). While BMDCs showed CPE even when STAT-1 was present, wild-type (wt) BMMΦ exhibited less CPE than their STAT1^−/−^ counterparts. These data showed that MNV-1 had a marked tropism for MΦ and DCs but not fibroblasts.

We used this information to screen available MΦ cell lines for growth of MNV-1, including the murine lines RAW 264.7 (Raschke et al. 1978) and J774A.1 (Ralph et al. 1975), and the human/murine hybrid line WBC264-9C (Aksamit 1986). These cells also showed visible CPE when inoculated with the MNV-1 stock (Figure 2, data not shown). Plaques were observed when infected RAW 264.7 monolayers were maintained under agarose (Figure 2A), allowing us to develop a plaque assay and quantitate virus titers.

STAT1^−/−^ BMMΦ, STAT1^−/−^ and wt BMDCs, and RAW 264.7 cells consistently supported the growth of MNV-1, while wt BMMΦ varied in their ability to support virus growth (Figure 2B). BMMΦ and BMDCs cells lacking STAT-1 always yielded higher MNV-1 titers than their wt counterparts. Furthermore, a low level of virus replication was observed in STAT1^−/−^ MEFs, but as reported previously, no virus growth was observed in wt MEFs (Karst et al. 2003). MNV-1 replication proceeded rapidly in permissive cells, with newly synthesized infectious virions first detected in cell lysates 9 to 12 hours postinfection (h.p.i.). Taken together, these data indicated that MNV-1 could productively infect MΦ and DCs.

### Verification of Viral Growth In Vitro

**Figure 3:**
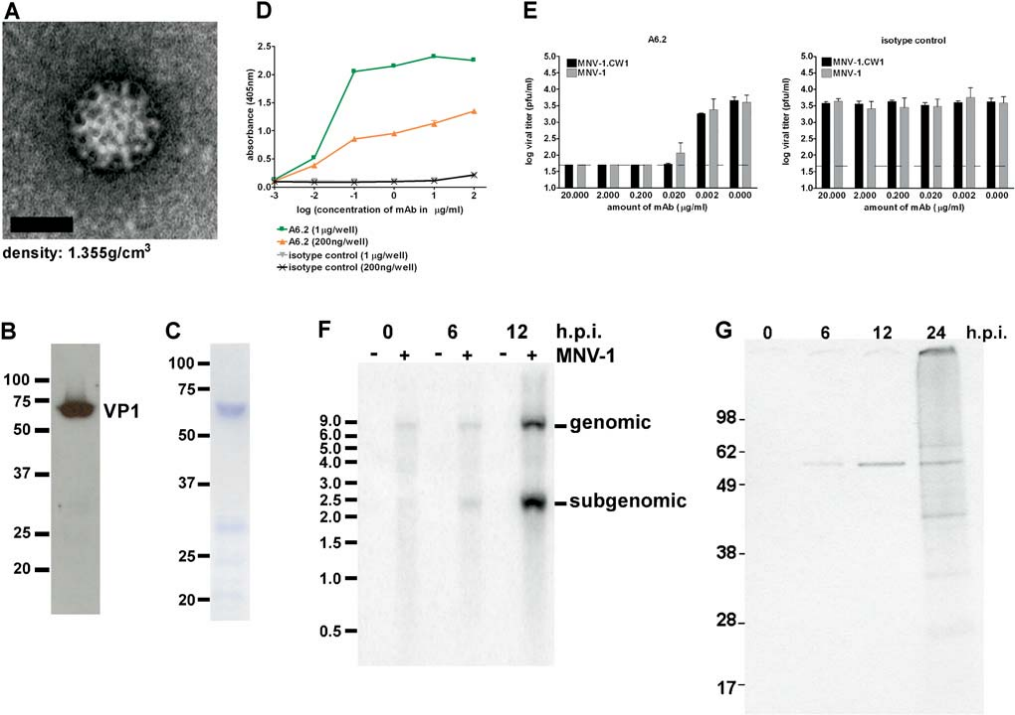
Characterization of the Triple Plaque-Purified Strain MNV-1.CW1 (A–C) MNV-1.CW1 purified on CsCl density gradients was visualized by (A) negative staining electron microscopy, (C) Coomassie staining, and (B) Western blot analysis with a polyclonal anti-MNV-1-capsid antibody. Molecular weight markers are indicated in kiloDaltons. (D) Specific binding of mAb A6.2 to two different concentrations of CsCl-purified MNV-1 particles in an enzyme-linked immunosorbent assay. (E) Neutralization of MNV-1 from brain homogenate and MNV-1.CW1 by mAb A6.2 but not the isotype control (10H2) mAb in a plaque neutralization assay. The assay was repeated three times to calculate standard deviations. The limit of detection is indicated by the dashed line. (F) Timecourse of viral RNA synthesis in RAW 264.7 cells. Northern blot analysis of viral RNA from cells infected with MNV-1.CW1 (MOI of 2.0) or mock-infected cells. The size of RNA markers in kilobases is shown on the left. The positions of subgenomic- and genomic-length RNA are indicated on the right. This timecourse is a representative of two independent experiments. (G) Timecourse of viral protein synthesis in infected RAW 264.7 cells. MNV-1-specific proteins were precipitated from radiolabeled cell lysates of MNV-1.CW1-infected RAW 264.7 cells (MOI of 2.0) at indicated times after infection. The size of the proteins in kiloDaltons is indicated.

Several approaches were used to verify that the observed CPE and plaques were caused by MNV-1. We first performed a clonal selection from the MNV-1 stock (from infected brain tissue) with three rounds of plaque purification in RAW 264.7 cells to generate the MNV-1.CW1 strain. This strain was amplified in RAW 264.7 cells, after which virus particles were concentrated and subjected to purification by isopycnic centrifugation in CsCl. A distinct band was observed in CsCl gradients at a density of 1.35 ± 0.01 g/cm^3^, consistent with that described for noroviruses (Kapikian et al. 1996). Examination of the material in this fraction by negative staining electron microscopy showed the presence of virus particles with calicivirus morphology (Figure 3A). Furthermore, SDS-PAGE analysis of this material revealed a major protein of approximately 59 kDa, consistent with the calculated mass of the MNV-1 capsid protein (Figure 3B,C). Western blot analysis with antibodies generated against bacterially expressed MNV-1 capsid protein (Figure 3B) and mass spectrometry (data not shown) confirmed its identity as the MNV-1 capsid protein. A genomic-sized RNA molecule of approximately 7.4 kb was detected in nucleic acid isolated from the purified virions with a probe specific for the MNV-1 genome in Northern blots (data not shown). Finally, a neutralization assay was performed with the monoclonal antibody (mAb) A6.2 specific for the MNV-1 capsid protein (see Materials and Methods). MAb A6.2 specifically bound to CsCl-purified MNV-1 virions in an immunoassay, while the isotype-matched mAb 10H2, an anti-reovirus μ1c mAb (Virgin et al. 1991), did not bind (Figure 3D). MAb A6.2, but not the isotype control antibody 10H2, showed neutralization activity in a plaque reduction assay for both the virus in the original brain homogenate (MNV-1), and the three-times plaque-purified strain MNV-1.CW1 (Figure 3E). Together these data confirmed that MNV-1 was the infectious agent associated with viral growth observed in the infected cell cultures.

### MNV-1 RNA and Protein Production in Permissive Cells

To compare MNV-1 replication in cells with that of other caliciviruses, we analyzed viral RNA and protein synthesis in MNV-1.CW1-infected RAW 264.7 cells. Northern blot analysis using a probe specific for the positive strand of the MNV-1 genome showed an increase in the accumulation of full-length (7.4 kb) and subgenomic-length (2.3 kb) MNV-1 genome over time (Figure 3F). Radiolabeled MNV-1-infected RAW 264.7 cell lysates were analyzed by immunoprecipitation with serum from a MNV-1 infected mouse, and a 59-kDa protein consistent with the capsid protein was detected as early as 6 h.p.i. (Figure 3G). Additional proteins accumulated over time that corresponded in size to expected calicivirus nonstructural proteins such as the 76-kDa proteinase-polymerase precursor and an approximately 40-kDa NTPase protein (Sosnovtsev et al. 2002). These data showed that the viral RNA and proteins synthesized in infected cells were consistent with calicivirus replication (Green et al. 2001).

### Ultrastructural Examination of MNV-1-Infected RAW 264.7 Cells

**Figure 4:**
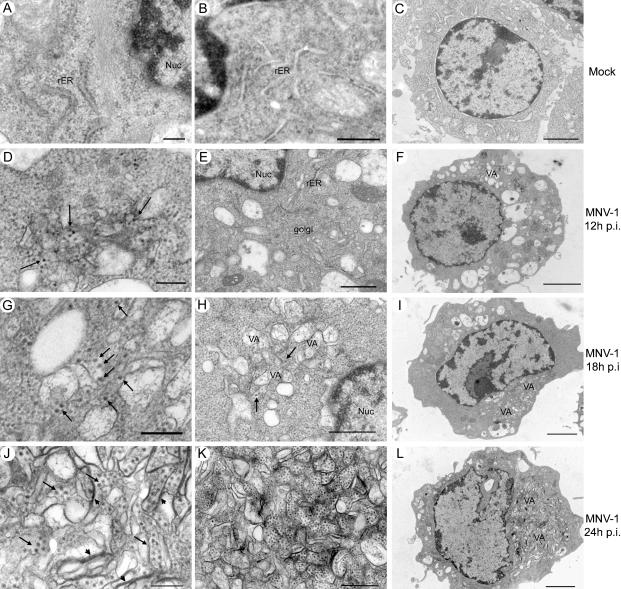
Ultrastructural Studies of MNV-1.CW1-Infected RAW 264.7 Cells Cells were infected with MNV-1.CW1 (P3) (MOI of 2.0) (D–L) or mock-infected (A–C) and processed for electron microscopy 12 (D–F), 18 (G–I), or 24 (A–C; J–L) h.p.i. MNV-1 particles are indicated by arrows and confronting membranes by arrowheads. VA, vesiculated areas; Nuc, nucleus; rER, rough endoplasmic reticulum. Scale bars, 200 nm for (A), (D), (G), and (J); 500 nm for (B), (E), (H), and (K); 2 μm for (C), (F), (I), and (L).

Positive-strand RNA viruses (Dales et al. 1965; Mackenzie et al. 1999; Pedersen et al. 1999), including caliciviruses (Love et al. 1975; Studdert and O'Shea 1975; Green et al. 2002), are known to replicate in association with intracellular membranes. Therefore, we examined the ultrastructural morphology of MNV-1.CW1-infected RAW 264.7 cells (Figure 4). Over time, virus-infected cells showed a striking change in overall morphology and intracellular organization (Figure 4D–4L) compared to mock-infected cells (Figure 4A–4C). Structures resembling virus particles were observed within or next to single- or double-membraned vesicles in the cytoplasm by 12 h.p.i. (Figure 4D). The vesiculated areas increased in size with time (Figure 4G–4I), and by 24 h.p.i., large numbers of these vesicles and viral particles occupied most of the cytoplasm, displacing the nucleus (Figure 4J–4L). In addition, a complete rearrangement of intracellular membranes with some confronting membranes occurred (Figure 4J), leading to a rearrangement of the endoplasmic reticulum and loss of an intact Golgi apparatus (Figure 4E; data not shown). Interestingly, these smooth-membraned vesicles were often surrounded by mitochondria. A small proportion of cells also showed crystalline arrays of cytoplasmic virus particles (data not shown). These observations indicate that like other positive-strand RNA viruses, norovirus RNA replication likely occurs in association with intracellular membranes.

### Characterization of the Plaque-Purified Strain MNV-1.CW1 In Vitro

**Figure 5:**
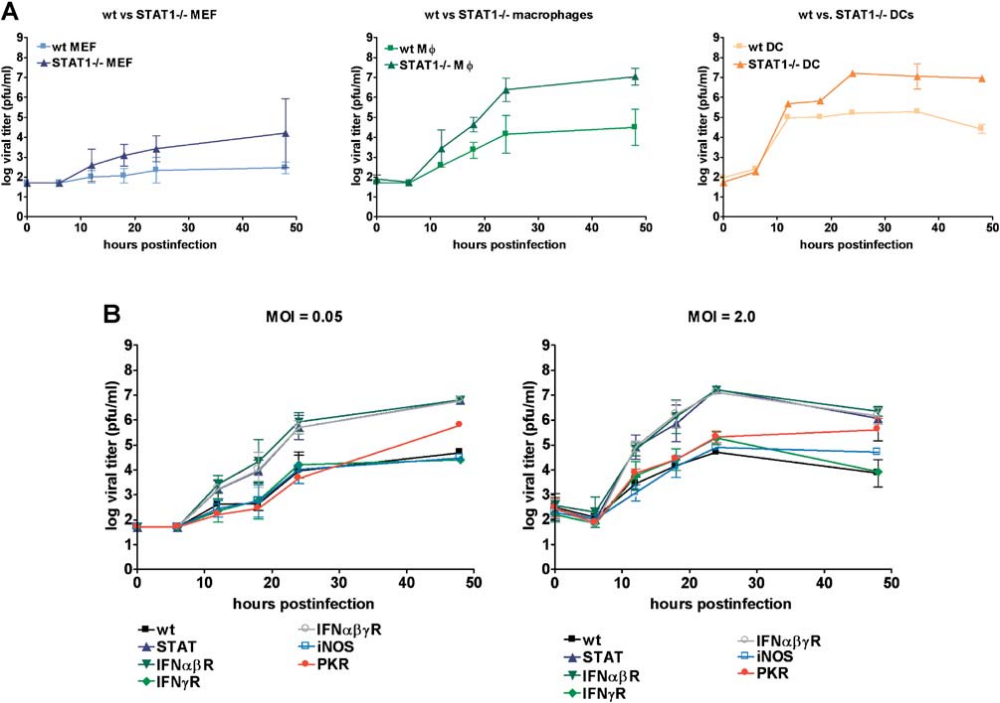
Critical Role for STAT-1 in Limiting MNV-1 Growth In Vitro (A) MNV-1.CW1 has no defect in viral growth in vitro. Growth curves (MOI of 0.05) were performed two or three times with MNV-1.CW1 (P3) on indicated cells to calculate standard deviations. (B) MNV-1 growth in MΦ is controlled by STAT-1 and Type I IFNs. BMMΦ of the indicated genotype were infected with MNV-1.CW1 (P3) at the indicated MOI. The experiment was performed twice to calculate standard deviations. The *p*-values for PKR versus wt infection at MOI 0.05 and 2.0, 0.8867 and 0.1616, respectively, are not significant. Statistical analysis was performed using the paired *t*-test (GraphPad Prism, version 3.03).

To determine whether the plaque purification and sequential amplification of MNV-1 in RAW 264.7 cells had altered its growth characteristics, different cell types were infected with passage (P) 3 of MNV-1.CW1. In general, the growth of MNV-1.CW1 (P3) in wt or STAT1^−/−^ MΦ and MEFs (Figure 5A) as well as RAW 264.7 cells (data not shown) was similar to that observed for the original parental MNV-1 virus stock (compare Figure 2B and 5A). Virus titers were reproducibly higher in STAT1^−/−^ cells compared to wt cells, and MNV-1.CW1 (P3) growth was consistently observed in wt BMMΦ. These data demonstrated that our plaque purification and serial passage in RAW 264.7 cells had not changed the tropism of the virus for primary DCs and MΦ and confirmed the importance of STAT-1 in controlling MNV-1 growth at the cellular level.

### Cellular Factors Controlling MNV-1 Growth In Vitro

Previous studies demonstrated that a lack of STAT-1 or both IFNαβR and IFNγR increase susceptability to MNV-1 infection. Mice lacking individual IFNR, inducible nitric oxide (iNOS)^−/−^, or protein kinase R (PKR)^−/−^ are not susceptible (Karst at al. 2003). Therefore, we determined whether molecules other than STAT-1 exhibited antiviral effects at the level of the infected cell. Primary BMMΦ from wt mice or mouse strains deficient in STAT-1, IFNαβR, IFNγR, IFNαβγR, iNOS, or PKR were directly compared for their ability to support virus replication at two different multiplicities of infection (MOIs) (Figure 5B). Again, BMMΦ cells from both wt and STAT1^−/−^ mice supported MNV-1 virus replication, with higher titers observed in cells deficient in STAT-1. Cells obtained from mice lacking both Type I and II IFNR (IFNαβγR^−/−^) or Type I IFNR alone (IFNαβR^−/−^) supported replication of virus as efficiently as STAT1^−/−^ cells. In addition, wt BMMΦ and wt BMDCs secrete IFNα after MNV-1-infection, as determined by IFNα enzyme-linked immunosorbent assay (ELISA) (data not shown). This is consistent with a direct role for IFN signaling in MNV-1 growth but does not rule out the possibility that effects of STAT-1 and IFNαβR occur in vivo prior to explantation of the bone marrow. Absence of IFNγR, iNOS, or PKR did not have a statistically significant effect on MNV-1 growth in BMMΦ. Together, these data demonstrate that the antiviral molecules STAT-1 and IFNαβ are part of a cellular response that limits norovirus growth.

### Characterization of the Plaque-Purified Strain MNV-1.CW1 In Vivo

**Figure 6:**
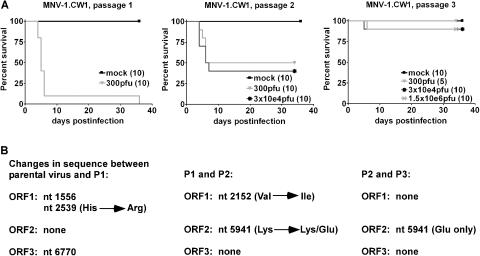
Changes in Virulence of Plaque-Purified MNV-1 over Multiple Passages Are Associated with Limited Amino Acid Changes (A) Serial passage of MNV-1.CW1 in cell culture causes attenuation. STAT1^−/−^ mice were infected orally with the indicated virus dose. The number of mice analyzed is indicated in parentheses. (B) Summary of sequence analysis of MNV-1 over several passages. The nucleotide and amino acid differences between the indicated viruses are shown (for detail see Table 1).

To address the effects of cell culture adaptation on virulence, STAT1^−/−^ mice were infected orally with MNV-1.CW1 from three successive passages (P1, P2, and P3) (Figure 6A). Oral administration of MNV-1.CW1 (P1) resulted in lethal infection, similar to that previously reported for the parental MNV-1 brain tissue stock (Karst et al. 2003). These data fulfill a Koch's postulate with regard to MNV-1 infection and are consistent with the identification of MNV-1 as the infectious agent that was passaged in animals in our initial studies (Karst et al. 2003). In contrast, MNV-1.CW1 (P3) failed to cause a lethal infection in STAT1^−/−^ mice after oral inoculation, even when administered a dose of 1.5 × 10^6^ plaque-forming units (pfu), 5,000 times greater than the lethal dose for P1. In addition, immunohistochemical analysis of sectioned spleen and liver from STAT1^−/−^ mice infected orally with 1.5 × 10^6^ pfu of MNV-1.CW1 (P3) did not reveal any MNV-1-specific staining, unlike the parental virus (see Figure 1, data not shown). This striking difference in virulence and decrease of viral antigen in infected mice, coupled with an intermediate lethality phenotype of the MNV-1.CW1 (P2) virus, showed that serial passage of the virus in cell culture could attenuate MNV-1 virulence in vivo.

**Table 1:**
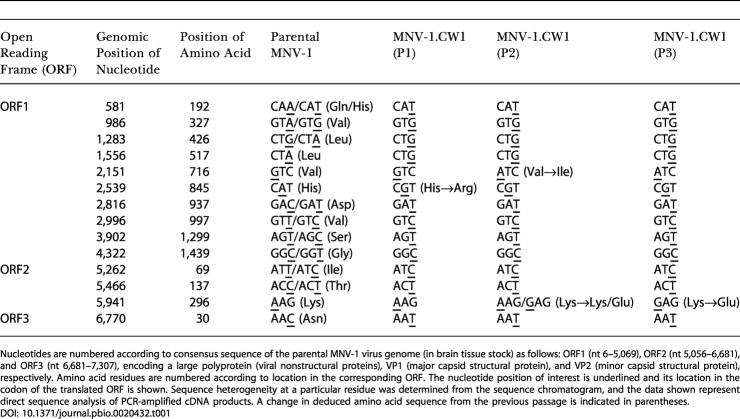
Sequence Analysis of MNV-1 over Several Passages

Nucleotides are numbered according to consensus sequence of the parental MNV-1 virus genome (in brain tissue stock) as follows: ORF1 (nt 6–5,069), ORF2 (nt 5,056–6,681), and ORF3 (nt 6,681–7,307), encoding a large polyprotein (viral nonstructural proteins), VP1 (major capsid structural protein), and VP2 (minor capsid structural protein), respectively. Amino acid residues are numbered according to location in the corresponding ORF. The nucleotide position of interest is underlined and its location in the codon of the translated ORF is shown. Sequence heterogeneity at a particular residue was determined from the sequence chromatogram, and the data shown represent direct sequence analysis of PCR-amplified cDNA products. A change in deduced amino acid sequence from the previous passage is indicated in parentheses

### Molecular Analysis of Serially Passaged MNV-1.CW1

To examine the molecular basis for this attenuation, consensus sequence analysis was performed on the RNA genome of MNV-1 present in the original brain tissue stock (parental virus), and in viruses from each subsequent cell culture passage of MNV-1.CW1 (P1 through P3) (Figure 6B; Table 1). Three nucleotide changes occurred between the parental virus and P1, with one of these resulting in an amino acid substitution (histidine to arginine) at residue 845, located within the predicted “3A-like” region of the nonstructural polyprotein. In the P2 virus, which retained virulence but at a reduced level compared to the parental and P1 viruses, a second nucleotide substitution within the predicted “3A-like” coding region was observed that caused an amino acid change (valine to isoleucine) at residue 716. The partial attenuation of virulence of the P2 virus in vivo is of interest since the homologous protein in poliovirus, the 3A protein, alters the amount of cytokines secreted from cells, with likely effects on viral pathogenesis (Dodd et al. 2001). Of note, a mixed population of A and G nucleotides was detected at position 5,941 of the P2 viral genome that could potentially yield two populations of virus with either amino acid lysine or glutamic acid at residue 296 of the capsid protein. In the P3 virus, which was avirulent in mice, the G nucleotide sequence at position 5,941 emerged as the predominant sequence. The resulting amino acid substitution was of interest because of its location within the hypervariable P2 domain, which contains the putative receptor-binding site (Prasad et al. 1994, 1999; White et al. 1996). However, altered virus binding to permissive cells cannot explain the attenuated phenotype since the parental virus and MNV-1.CW1 (P3) replicate to similar levels in BMDCs and BMMΦ in vitro. Similar to our findings, the P2 domain was also implicated in attenuation of porcine enteric calicivirus virulence (Guo et al. 2001). This study suggests that the norovirus capsid protein, especially the hypervariable P2 domain, and possibly the 3A-like protein, may be important sites for the development of virulence-attenuating mutations.

### Conclusion

Detection of MNV-1-positive cells of the MΦ and DC lineage in infected organs of STAT1^−/−^ mice led to our finding that MNV-1 grows in these cell types in vitro. This provides the first tissue culture model for a norovirus. In addition, the antiviral Type I IFN response with signaling through STAT-1 is crucial for resistance to murine norovirus infection in vivo and in vitro. Taken together the previous in vivo data (Karst et al. 2003) and the tropism of this norovirus for cells of the innate immune system, underscore the importance of the innate immune response, specifically STAT-1 and Type I IFNs, in resistance against norovirus infection. These data may aid the development of a culture system for human noroviruses since neither cells of the MΦ/DC lineage nor cells with defects in the Type I IFN/STAT-1 antiviral pathway have likely been investigated (Duizer et al. 2004). Furthermore, this MNV-1 tissue culture model will help elucidate stages of the viral life cycle and cellular factors essential for norovirus replication that may provide targets for prevention or control of an important human disease.

The demonstration of a tropism of MNV-1 for DCs was unexpected, as a calicivirus tropism for DCs has not been previously described. However, like MNV-1, other caliciviruses do interact with MΦ. Viral RNA from rabbit hemorrhagic disease virus, a lagovirus, was detected in splenic and alveolar MΦ by in situ hybridization (Kimura et al. 2001). In addition, feline calicivirus, a vesivirus, showed a small, transient increase in viral titers in alveolar MΦ cultures, indicative of abortive infections (Langloss et al. 1978). It is possible that MΦ contribute to the spread of the virus through the host, but it must be noted that MΦ supported MNV-1 growth to a lower extent than DCs unless they lacked specific immune defense molecules such as STAT-1. This argues that MΦ may be the cell through which STAT1-dependent innate immunity limits MNV-1 virulence (Karst et al. 2003).

In contrast to MΦ, DCs were permissive even when STAT-1 was present (see Figure 2). DCs are sentinels of the immune system whose function is to acquire antigens and stimulate lymphocytes. Intestinal DCs are found in the gut in specialized lymphoid tissues where they can sample enteric antigens by extending their dendrites into the gut lumen (Stagg et al. 2003; Kraehenbuhl and Corbett 2004). We therefore speculate that DCs in humans and mice provide noroviruses access to subepithelial regions of the intestine, thereby contributing to norovirus disease pathogenesis. Further studies are in progress to address the role of MΦ and DCs in the intestine and the physiologic relevance of these cells for MNV-1 pathogenesis in general.

## Material and Methods

### 

#### Cell cultures and mice

MEFs were generated and cultured as described previously (Pollock et al. 1997). RAW 264.7 cells were purchased from ATCC (Manassas, Virginia, United States) and maintained in DMEM (Cellgro, Mediatech, Herndon, Virginia, United States) supplemented with 10% low-endotoxin fetal calf serum (SH30070.03, HyClone, Logan, Utah, United States), 100 U penicillin/ml, 100 μg/ml streptomycin, 10 mM HEPES (N-2-hydroxyethylpiperazine-N′-2-ethanesulfonic acid), and 2 mM L-glutamine (Biosource, Camarillo, California, United States). Bone marrow was harvested and MΦ were cultured as described previously (Heise et al. 1998). To culture DCs, bone marrow cells were resuspended in RPMI1640 containing 10% low endotoxin fetal calf serum, 2 mM L-glutamine, 1 mM sodium pyruvate (Biosource), 100 U penicillin/ml, 100 μg/ml streptomycin, 1% nonessential amino acids (Biosource), and 20 ng/ml recombinant mouse GM-CSF (BD Biosciences, San Jose, California, United States) and plated at a concentration of 3 × 10^5^ cells/ml in six-well plates in a total volume of 3 ml per well. The percentage of CD11c-positive DCs was determined by FACS staining after culturing cells for 7 d at 37 °C and 5% CO_2_. Approximately 70% of the cells were CD11c positive.

Wt 129 and STAT1^−/−^ mice were purchased from Taconic (Germantown, New York, United States). IFNαβR^−/−^, IFNγR^−/−^, and IFNαβγR^−/−^ (Muller et al. 1994), PKR^−/−^ (Yang et al. 1995), and iNOS^−/−^ (MacMicking et al. 1995) mice were bred and housed at Washington University in accordance with all federal and university policies.

#### Preparation of rabbit anti-MNV-1 serum

Rabbits were immunized subcutaneously with 140 μg of MNV-1 VLPs in complete Freunds adjuvant and boosted 4 or 8 wk later with 70 μg of MNV-1 VLPs or 50 μg of UV-inactivated CsCl-purified MNV-1 in incomplete Freunds adjuvant. Serum was collected two weeks after the last boost, heat inactivated, and filtered before use.

#### Immunohistochemistry

Seven-week-old STAT1^−/−^ mice were infected orally with 25μl of brain homogenate containing MNV-1 (6 × 10^5^ pfu) or brain homogenate from uninfected mice. Organs were collected into 10% buffered formalin and embedded in paraffin for sectioning by standard methods. Immunohistochemistry was performed as described previously (Weck et al. 1997) using tyramide signal amplification (NEN Life Science Products, Boston, Massachusetts, United States). Slides were blocked in tyramide signal amplification blocking reagent (NEN Life Science Products) containing 10% mouse serum (IHC blocking buffer) for 30 min before adding antibodies. Serum was diluted 1:20,000 (spleen) or 1:100,000 (liver) in IHC blocking buffer, and tissue sections were incubated overnight at 4 °C. Horseradish peroxidase–conjugated donkey anti-rabbit secondary antibody (Jackson ImmunoResearch Laboratories, West Grove, Pennsylvania, United States) was diluted 1:250 in IHC blocking buffer and applied to tissue sections for 1 h at room temperature. Biotin-tyramide was added at a dilution of 1:50 in 1× amplification diluent (NEN Life Science Products) for 10 min, slides were washed, and horseradish peroxidase–conjugated streptavidin (NEN Life Science Products) was added at a 1:100 dilution in tyramide signal amplification blocking reagent and incubated for 30 min at room temperature before washing. Antigen was visualized by a 3-min staining with a solution of 3, 3′-diaminobenzidine (Vector Laboratories, Burlingame, California, United States). Slides were washed and lightly counterstained with hematoxylin, dehydrated, and covered with Cytoseal XYL (Richard Allan Scientific, Kalamazoo, Michigan, United States) coverslips. No staining was observed in infected tissues incubated with preimmune serum or mock-infected tissues incubated with immune serum.

#### Infection of cells

Adherent cells were plated in 12-well plates and allowed to attach for several hours. Infections were carried out at an MOI of 0.05 or 2.0 for 30 min on ice in a volume of 0.5 ml per well. DCs were infected in bulk in the same volume. Cells were then washed twice with 2 ml of ice-cold PBS per well. To allow viral entry, 1 ml of medium was added to each well, and cells were incubated at 37 °C and 5% CO_2_ for different time periods. For growth curve samples, infected cells and media were subjected to two or three cycles of freezing and thawing before plaque titration.

#### Generation of mAb A6.2

A MNV-1-seropositive 129 mouse was injected intraperitoneally with 100 μl of a brain homogenate containing MNV-1, and the spleen was harvested 3 d later. Hybridoma fusions were performed as described previously (Virgin et al. 1991) with the following modifications. Hybridoma supernatants were screened for binding to recombinant MNV-1 capsids by ELISA as described (Karst et al. 2003). Stable hybridomas were characterized by Western blotting and ELISA after two rounds of subcloning by limiting dilution. A6.2 was unable to detect MNV-1 capsid protein by Western blot analysis but specifically bound to recombinant MNV-1 capsids by ELISA. The A6.2 isotype is IgG2a and was determined using the mouse mAb isotyping kit (Amersham Biosciences, Amersham, United Kingdom) and following manufacturer's protocol.

#### MNV-1 plaque assay and plaque neutralization assay

RAW 264.7 cells were seeded into six-well plates at a density of 2 × 10^6^ viable cells per well. On the following day, 10-fold dilutions of virus inoculum were prepared in complete DMEM medium and plated in duplicate wells. Plates were incubated for 1 h at room temperature on a rocking apparatus before aspirating the inoculum and overlaying the cells with 2 ml of 37–40 °C 1.5% SeaPlaque agarose in MEM supplemented with 10% low-endotoxin fet al.calf serum, 1% HEPES, 1% penicillin/streptomycin, and 2% glutamine (complete MEM) per well. Plates were incubated at 37 °C and 5% CO_2_ for 2 d. To visualize plaques, cells were stained with 2 ml of 56 °C 1.5% SeaKem agarose in complete MEM containing 1% neutral red per well for 6–8 h.

For plaque neutralization assays, differing concentrations of purified mAb (A6.2, anti-MNV-1 capsid; isotype control, 10H2, anti-reovirus μ1c) were incubated with equal plaque-forming units of either MNV-1.CW1 or MNV-1 brain homogenate for 30 min at 37 °C prior to performing the MNV-1 plaque assay.

#### Purification of virus particles

RAW 264.7 cells were infected with MNV-1.CW1 for 2 d at an MOI of 0.05. Cellular debris was removed from freeze/thaw lysates by low-speed centrifugation for 20 min at 3,000 rpm. Supernatants were layered on top of a 5-ml 30% sucrose cushion and centrifuged at 4 °C for 2.5 h at 27,000 rpm (90,000 *g*) in a SW32 rotor. Cell pellets were then resuspended in PBS and mixed with CsCl to a final density of 1.335 g/cm^3^ and centrifuged for at least 18 h at 35,000 rpm (115,000 *g*) in a SW55 rotor. A wide lower band (1.35 ± 0.01g/cm^3^) and narrow upper band (1.31 ± 0.01g/cm^3^) were typically seen in the gradient. Each band was collected by puncturing the side of the tube with a needle before overnight dialysis against PBS at 4 °C.

#### Protein analysis

CsCl-purified virions were separated by SDS-PAGE gel electrophoresis using standard protocols (Sambrook et al. 1989). Proteins were visualized by Coomassie blue staining using the Simply Blue safe stain (Invitrogen, Carlsbad, California, United States) according to manufacturer's instructions. For Western blot analysis, proteins were transferred to nitrocellulose membrane and incubated with an anti-MNV-1-capsid rabbit polyclonal antibody, followed by a peroxidase-labeled secondary antibody, and visualized by ECL (Amersham Biosciences) according to manufacturer's instructions. Immunoprecipitation of radiolabeled infected cell lysates was performed as described previously (Sosnovtsev et al. 2002) with serum obtained from a 129 wt mouse infected orally with MNV-1.

#### Northern blotting

The region of the MNV-1 genome from nt 5,617 to 7,039 was amplified by RT-PCR and cloned into the pGEM-T Easy (Promega, Madison, Wisconsin, United States) vector between the T7 and SP6 promoters. The resulting plasmid was linearized with Bsu361 and in vitro transcribed with SP6 RNA polymerase (Roche, Indianapolis, Indiana, United States) to generate RNA transcript probes for detection of positive-sense viral RNA, or with T7 polymerase (Roche) to generate transcripts for detection of negative-sense viral RNA. To label probes, the transcription reaction was carried out in the presence of [P^32^]-UTP according to manufacturer's recommendations. Total RNA from virus-infected or mock-infected cells was isolated using Trizol (Invitrogen) according to the manufacturer's recommendations. One microgram of total RNA from MNV-1- or mock-infected cells was subjected to electrophoresis on a 1% formaldehyde gel. RNA Millennium Size Markers (Ambion, Austin, Texas, United States) were used as size markers. Northern blotting was performed using standard protocols (Sambrook et al. 1989). Probes were hybridized overnight at 68 °C in 50% formamide containing 6× SSC, 5× Denhardt's, 0.5% SDS, and 100 μg/ml ssDNA.

#### MNV-1 ELISA

The ELISA was performed as described previously (Karst et al. 2003) with the following modifications. ELISA plates were coated overnight at 4 °C with CsCl-purified MNV-1 particles at 0.2 or 1.0 μg/well. Diluted purified anti-MNV-1-capsid (A6.2) and isotype control (reovirus 10H2) mAbs, as well as the peroxidase-labeled secondary antibodies, were incubated for 60 min at 37 °C.

#### Electron microscopy

Negative staining electron microscopy of CsCl-purified virions was performed as described previously (Karst et al. 2003). For thin-section electron microscopy, RAW cells were infected with MNV-1.CW1 at an MOI of 2.0, as described above. At various times postinfection cells were washed with PBS and fixed with 3% glutaraldehyde diluted in PBS at room temperature for 2 h. Cells were pelleted and washed with buffer prior to incubation with 1% osmium tetroxide (in 0.1 M cacodylate buffer) for 40 min at room temperature. After washing, the cells were incubated overnight at 4 °C in 2% uranyl acetate/80% acetone. The pellets were then dehydrated with an acetone series and embedded in Epon before polymerization at 65 °C for 72 h. Ultrathin sections (60 nm) were cut with a Micro Star (Huntsville, Texas, United States) diamond knife, and the sections were stained and contrasted with uranyl acetate and lead citrate before viewing on a JOEL 1010 electron microscope at 80 kV. Images were captured on a MegaView III side-mounted CCD camera (Soft Imaging System, Lakewood, Colorado, United States), and figures were processed using Adobe Photoshop software (Adobe Systems, San Jose, California, United States).

#### Consensus sequence analysis of viral RNA

RNA was extracted from brain tissue or cell culture material with Trizol (Invitrogen) and reverse transcribed with Superscript II enzyme (Invitrogen). Genome-specific sequences were PCR-amplified with Elongase enzyme (Invitrogen) to produce seven overlapping fragments. The DNA fragments were gel-purifed and sequenced directly with reagents in the BigDye Terminator version 3.1 Cycle Sequencing Kit (Applied Biosystems, Foster City, California, United States) on a 3100 DNA sequencer (Applied Biosystems). Data were analyzed with the Sequencher software package (Gene Codes Corporation, Ann Arbor, Michigan, United States). Oligonucleotide primer sequences are available upon request.

## Abbreviations

BMDC: bone-marrow-derived dendritic cell
BMMΦ: bone-marrow-derived macrophage(s)
CPE: cytopathic effect
DC: dendritic cell
ELISA: enzyme-linked immunosorbent assay
h.p.i.: hours postinfection
IFN: interferon
IFN[αβγ]R: interferon[αβ] receptor and interferon[γ] receptor
iNOS: inducible nitric oxide
mAb: monoclonal antibody
MΦ: macrophage(s)
MEF: murine embryo fibroblast
MNV-1: murine norovirus 1
MOI: multiplicity of infection
ORF: open reading frame
P: passage
pfu: plaque-forming units
PKR: protein kinase R
wt: wild-type
